# Garden Cities for Consumptives

**Published:** 1913-11-01

**Authors:** J. E. Esslemont


### GARDEN CITIES FOR CONSUMPTIVES.

### A Plea for a National Scheme.

By J. E. ESSLEMONT, M.B.

The object of this paper is to bring forward a scheme by which I hope the complete eradication of tuberculosis may be brought within the sphere of "practical politics." Put briefly, the idea is to carry out the segregation of infectious cases in such a way as to afford complete safety from infection to the general public, while at the same time enabling the patients to continue such work as they are capable of and to enjoy the best possible chance of recovery and the fullest and most varied life which their condition allows. I believe that by the establishment of large colonies for consumptives on garden-city lines, this may be achieved with the minimum of expense and the maximum of benefit to both public and patients. For very advanced cases hospital or sanatorium accommodation where the patients can be nursed and made as comfortable as possible is all that is required, but for the much larger class of patients, still fit for a certain amount of work, sanatoria do not meet the case. It is only rarely that a patient can be cured in three or six months and safely return to his old surroundings. In the majority of cases he must live a suitable open-air life for years, or for the rest of his days, if he is to get well and keep well. Sanatorium life is too limited and monotonous and too expensive to be kept up for the necessary length of time, and for the proper treatment of these cases I see no alternative to a colony scheme. At least 100,000 patients will have to be provided for, so it is imperative that the scheme shall be economical as well as efficient. The colonies should be large, in order to provide sufficient variety of work, of recreation, and of scenery. I imagine something on the scale of Letchworth Garden City, a central town surrounded by a belt of agricultural land, the whole covering an area of a few square miles and capable of accommodating a population of 20,000 or 30,000. Smaller experimental colonies would probably be necessary to begin with, but mere farm colonies will not answer the purpose. Factory hands and others accustomed to the bustle and variety of a large town will not, as a rule, settle down to humdrum life on a farm, and for the majority of cases farm work is unsuitable. A town is essential if we are to provide for town-dwellers—a garden city without slums and overcrowding and the other evils of ordinary towns, but with all the advantages of division of labour, of convenience, and of social intercourse and enjoyment which only a town can offer. Sanatoria and farm colonies are no doubt very useful institutions and have done much to prepare the way for a larger scheme, but what I wish to make clear is that for the prevention of infection on a national scale they are utterly inadequate.

### Work in the Colony.

In such a colony patients could take part in innumerable kinds of work—farming, gardening, poultry farming, carpentry, industrial and clerical work, storekeeping, domestic service, etc. Many even of the more responsible posts could be held by patients, and work could be found for phthisical doctors, nurses, teachers, architects, engineers, and clergymen. It has been amply proved in many sanatoria, both in this country and abroad, that suitably graduated work is highly beneficial for non-febrile consumptive patients and far better for them than idleness. To get the best results, however, the work must be adapted to the patient's powers and of a nature interesting to him. It is surprising in many cases how strictly limited is the sphere in which a man can do his best work, and one of the great secrets of success in organising a community lies in finding the right sort of work for each to do. In many cases the patients would require to be trained to some work, and the industrial staff ought to include a sufficient number of experts capable of training learners in the best methods. The services of such experts would be remunerative, owing to the better quality of work done.

### The Question of Remuneration.

The amount of work done by each individual would be carefully regulated by the medical officers, patients working eight, six, four, or any smaller number of hours as prescribed, and attempting only such tasks as were approved of by the doctor. Time-tables would be arranged, as in sanatoria, so as to allow for suitable rest hours. The work would be organised throughout with a view to making the colonies, as far as possible, self-supporting. Most of the goods required could be produced within the colonies, and the production would be carried on, as far as possible, by the patients themselves, many of whom would be highly skilled workers. Great economies would thus be effected, and the cost of the colonies to the community would be reduced to a mere fraction of that entailed by treating a similar number of patients in sanatoria on present lines.

Patients would receive remuneration for their work, partly in board, lodgings, and treatment, partly in vouchers which could be exchanged for goods in the stores of the colony, and partly, so far as funds permitted, in cash, to enable them to carry on transactions with the general community outside. The voucher system, owing to the low cost of production within the colony and the elimination of trade expenses, would enable the workers to receive full value for their work at a much less cost than if they were paid entirely in cash.

It will be obvious that by such arrangements the economic value of the patients will be preserved to the fullest possible extent, and, as they will be paid for their work, there will be a strong inducement to them to enter the colony as soon as the disease is detected, in order to avail themselves of the exceptionally favourable conditions of work offered. This will have a double advantage, giving the patients a much better chance of recovery and bringing into the colony a large number of patients still fit for a good day's work, whose labour would therefore help to make it self-supporting. There are thousands of men and women in the country to-day in the earlier stages of consumption who would be quite able to do a fair amount of work under favourable circumstances, but who are not fit for a full day's work in the conditions under which they have to live at present. In the general community they are faced with the cruel alternatives of either sticking to work that is slowly killing them, or else giving up their jobs altogether. Usually, and especially if they have others dependent on them, they stick to their work, with disastrous results to themselves and at great risk to their neighbours, until absolutely compelled to give in. Others again who have been treated in sanatoria and have in a measure recovered their fitness for work, dread going back to the old surroundings in which they contracted the disease; dread, too, if they are conscientious, the possibility of passing on to others the disease from which they are suffering. For these people what could be more welcome than the establishment of a colony such as I am describing? I feel sure there would be no lack of applicants for admission, and each one admitted would not only get the best chance for himself, but would be one source of danger the less for the general community.

### The Architectural Aspect.

The site might consist partly of down or forest land, which would cost comparatively little, but should include a considerable proportion of grazing and arable land for the agricultural belt. The soil should be of a porous nature, and the site should be sheltered from rough rainy winds, which, as Dr. Gordon, of Exeter, has shown, seem to be particularly detrimental to phthisical patients. A good and abundant water supply would be essential, and a railway should run through the property.

Before building was commenced, a sketch plan of the whole colony would be prepared by an architect experienced in town-planning. The roads, railway sidings, sewerage system, and the various areas for factories, stores, residential quarters, open spaces, etc., would be mapped out. The residential buildings would consist partly of large sanatorium blocks accommodating 100 patients or more, partly of smaller houses and cottages, and of châlets, all adapted for open-air treatment. Blocks or groups of houses on the co-operative house-keeping system advocated by Ebenezer Howard, the founder of the garden-city movement, would probably find a place in the colony. Dwellings would be placed as far as possible on sunny, sheltered slopes commanding good open views.

Besides the residential quarters there would be administrative buildings, observation block, hospital, schools, stores, factories, workshops, farm-buildings, halls, churches, library, theatre, electric generating station, gas-works, laundry, etc.

All buildings would be constructed so as to afford the best possible conditions of light, air, and exposure, due regard being paid to strict economy and artistic appearance. An essential feature of the interiors would be the avoidance of all ridges, crevices, and corners where dust could accumulate. Smooth impervious surfaces and rounded angles, which could be easily and thoroughly cleansed and disinfected, would prevail throughout, in buildings, furniture, and fittings, and special care would be taken to leave no lurking places or breeding grounds in or about the buildings for flies, beetles, rats, mice, or other pests.

These special features in construction would apply, as far as possible, to every building in the colony, not only to the residential quarters, so that the colony would be unique in having even its factories, workshops, stores, and public buildings designed primarily with a view to health.

### Medical Treatment.

On arrival patients would remain for a few days in an observation block, where they would be thoroughly examined and their temperatures and other symptoms observed. Before they went farther it would be ascertained that they were not suffering from any other infectious disease or any condition which would make it undesirable that they should associate with the other patients. Their teeth would be examined and, if necessary, the services of the dentist would be called in. An estimate would be formed of the nature and amount of work each patient was fit for, and arrangements made for his accommodation and employment.

While the colonies would be designed primarily for those who were infectious but still able to work, it would be necessary to provide for patients at all stages, and also for a certain number of people who were unaffected by the disease—members of the staff or of patients' families. More or less separate provision would therefore have to be made for three classes:

(1) Healthy people and non-infectious cases,
(2) Infectious cases able to work, and
(3) Hospital cases, temporarily or permanently unfit for work.

Any patient belonging to the first or second class becoming temporarily incapacitated by a hæmorrhage, an attack of pleurisy, or other cause, would go to hospital until such time as he was again fit for work. The hospitals would always therefore contain a considerable proportion of curable cases, and no one would feel that being sent to hospital was equivalent to being condemned as incurable. In the case of patients able to work, medical supervision and control would be exercised much as in ordinary sanatoria.

In addition to treatment by means of open-air, graduated work and hygienic living, various special forms of treatment would be employed, and the colony would offer an unrivalled opportunity for deciding on the merits of different remedies, different dietaries, etc. Doctors and patients who were keen on trying any particular method of treatment would have the opportunity of doing so, and the success or failure of the experiment would be watched with interest by all. Research work on all matters connected with tuberculosis would be actively carried on.

The medical staff would include surgeons, dentists, throat specialist, radiographer, pathologist, bacteriologist, etc. The services of these specialists would be available for the examination or treatment of any case requiring them, and co-operation between the various members of the staff would result in a thoroughness of investigation and efficiency of treatment such as could not be attained in small institutions.

### Social and Family Arrangements.

It is when we come to social and family arrangements that the most debatable questions arise. The simple and drastic methods of segregation so successfully used in the case of cattle cannot be applied without modification where human beings are concerned. It would be impossible to insist on the ruthless and absolute separation of friends, of husband and wife, of parents and children, even in order to achieve the eradication of tuberculosis.

The admission of visitors to the colony and the occasional absence of patients on leave would have to be allowed, with such limitations and conditions as were necessary for the safety of the public. For cases in which a change of air and scene was desirable an interchange of patients between different colonies might be arranged.

In the case of married couples of whom one member was healthy and the other affected I think the healthy one should also be allowed to enter the colony if he or she elected to do so and were willing to conform to the necessary conditions. I think, however, that patients in an infectious stage of the disease should, as far as possible, be induced to regard themselves as unfit for parentage, and advised to lead a celibate life, and that the celibates should be taught that a chaste life is advisable for medical as well as moral reasons. Rigid separation of the sexes I do not consider advisable. On the contrary, it is desirable that the sexes should mingle quite freely at meal times and in their hours of work and recreation—just as freely as they do in well-regulated circles in the general community. I believe that such social intercourse is not only more natural and pleasant, but also better from the points of view of both health and morality.

The most difficult cases to deal with satisfactorily would be those in which either the breadwinner or the mother was affected, in a family which included young children. It would be unwise to lay down hard-and-fast rules for such cases. The interests of the children would have to be the paramount consideration, but in many cases it might be advisable to allow part or the whole of the family to enter the colony with the affected parent. Objections to such an arrangement will be dealt with later in the paper.

Each patient on entering the colony would receive instruction on the subject of tuberculous infection and the precautions necessary to prevent its spread. The colony would have a special code of morals on this subject, strict observance of which would be binding for all.

As has been mentioned, industrial training, when required, would also be a cardinal feature of life in the colony.

There would be open-air schools for children and institutions for the treatment and training of crippled children.

As regards recreation, there would be miles of country in which to ramble, while the less strenuous forms of sport and games would be permitted and provided for as far as possible. Clubs and societies of various kinds would naturally be formed, much as in the general community. There would also be musical and dramatic entertainments, etc. These and all religious services would be held either in the open air or in thoroughly ventilated buildings. It is essential that life should be made as enjoyable as possible for the patients. Happiness and hopefulness are unrivalled aids to health. It should not be forgotten, in our efforts to get rid of the contagion of disease, that there is such a thing as a contagion of health, which is of the utmost value. It would be a great mistake, even were it possible, to have a community composed entirely of consumptives in various stages. The presence among invalids of a certain proportion of healthy and vigorous people, with abundant vitality and of cheerful disposition, has a tonic influence without which it is impossible to get the best results. "A merry heart doeth good like a medicine, but a broken spirit drieth the bones."

### Business Organisation.

The trustees of the colony would retain sole control not only of the gas, water, and electric plants, but also of a large proportion of the residential quarters, farms, stores, factories, and workshops. Some of the more speculative kinds of business and industry might be left to private enterprise, which would, however, have to conform to such conditions as the Board of Trustees thought fit to impose. The colonies need not be reserved for the poor only, but might include sanatoria, nursing homes, or residences for paying patients. These would help to make the colonies self-supporting and might be staffed to a large extent by working patients.

The whole of the land would be vested in the trustees of the colony, but leases might be granted, on suitable terms and conditions, to private individuals, companies, or co-operative associations for either residential or business purposes. The development of the colony might be materially assisted by companies which, while not charitable institutions, would nevertheless have a philanthropic aim, and dividends restricted to a fixed percentage.

The administration of the colony would be in the hands of a Board of Trustees, which would contain representatives of the parent association of the colony (referred to later), or of the Treasury, Local Government Board, Insurance Authorities, County Councils, and others.

This Board might be assisted, as in the case of the Garden Village Trusts at Bournville and New Earswick, and the First Garden City Company at Letchworth, by a village or town council, which would be a consultative body elected by ballot of the inhabitants and dealing with matters affecting the interests and development of the colony, such as the public halls and recreation grounds, the public library, the encouragement of gardening and holding of flower shows, etc. The Board would also be assisted by a number of permanent committees—a finance committee, a building committee, a medical committee, etc. The administration would be carried out through a number of departmental staffs—administrative, medical, educational, agricultural, etc.

As in the case of the Welsh National Memorial scheme, a charter would be required, in order to confer on the trustees of the colonies the powers and privileges necessary for the success of the enterprise.

(*To be continued.*)

